# 2-O Heparan Sulfate Sulfation by Hs2st Is Required for Erk/Mapk Signalling Activation at the Mid-Gestational Mouse Telencephalic Midline

**DOI:** 10.1371/journal.pone.0130147

**Published:** 2015-06-15

**Authors:** Wai Kit Chan, Katherine Howe, James M. Clegg, Scott E. Guimond, David J. Price, Jeremy E. Turnbull, Thomas Pratt

**Affiliations:** 1 Centre for Integrative Physiology, The University of Edinburgh, Edinburgh, EH8 9XD, United Kingdom; 2 Centre for Glycobiology, Department of Biochemistry, Institute of Integrative Biology, The University of Liverpool, Liverpool, L69 7ZB, United Kingdom; Columbia University, UNITED STATES

## Abstract

Heparan sulfate (HS) is a linear carbohydrate composed of polymerized uronate-glucosamine disaccharide units that decorates cell surface and secreted glycoproteins in the extracellular matrix. In mammals HS is subjected to differential sulfation by fifteen different heparan sulfotransferase (HST) enzymes of which Hs2st uniquely catalyzes the sulfation of the 2-O position of the uronate in HS. HS sulfation is postulated to be important for regulation of signaling pathways by facilitating the interaction of HS with signaling proteins including those of the Fibroblast Growth Factor (Fgf) family which signal through phosphorylation of extracellular signal-regulated kinases Erk1/2. In the developing mouse telencephalon Fgf2 signaling regulates proliferation and neurogenesis. Loss of *Hs2st* function phenocopies the thinned cerebral cortex of mutant mice in which Fgf2 or Erk1/2 function are abrogated, suggesting the hypothesis that 2-O-sulfated HS structures play a specific role in Fgf2/Erk signaling pathway in this context *in vivo*. This study investigated the molecular role of 2-O sulfation in Fgf2/Erk signaling in the developing telencephalic midline midway through mouse embryogenesis at E12.5. We examined the expression of Hs2st, Fgf2, and Erk1/2 activity in wild-type and *Hs2st^-/-^* mice. We found that Hs2st is expressed at high levels at the midline correlating with high levels of Erk1/2 activation and Erk1/2 activation was drastically reduced in the *Hs2st^-/-^* mutant at the rostral telencephalic midline. We also found that 2-O sulfation is specifically required for the binding of Fgf2 protein to Fgfr1, its major cell-surface receptor at the rostral telencephalic midline. We conclude that 2-O sulfated HS structures generated by Hs2st are needed to form productive signaling complexes between HS, Fgf2 and Fgfr1 that activate Erk1/2 at the midline. Overall, our data suggest the interesting possibility that differential expression of Hs2st targets the rostral telencephalic midline for high levels of Erk signaling by increasing the sensitivity of cells to an Fgf2 signal that is rather more widespread.

## Introduction

Heparan sulfate proteoglycans (HSPGs) are cell surface and secreted molecules expressed by all animal cells. They consist of a core protein and covalently attached heparan sulfate (HS) carbohydrate side chains. HS is differentially sulfated by specific 2-O, 3-O, 6-O and N- sulfotransferases and 6-O desulfated by sulfatases giving rise to a potentially enormous variety of HS structures. This variety is key in HS’s biological role according to the HS “sugar code” hypothesis which states that different HS structures differentially instruct signaling pathways during biological processes including brain development [1–5]. Although it has been postulated for some time that HS regulates signaling pathways via interaction with signaling ligands and their receptors the molecular consequence of differential sulfation has only been studied in a few situations *in vivo* and very little is known about how differential HS sulfation functions molecularly in the developing brain [4,6–10].

Fgf2 is one of the fibroblast growth factor family of signaling ligands which signals through the Erk1/2 pathway to drive development [11]. In neurodevelopment, Fgf2 is required for cell proliferation and neurogenesis at early developmental stages [11–13]. There have been many *in vitro* and biochemical studies implicating HS in Fgf2 signaling. HS was shown to bind to Fgf2 and is needed for Fgf2 signaling to occur in cell lines and crystallography analysis confirmed the formation of a ternary structure between HS, Fgf2 and its receptor Fgfr1 [6–10,14]. Further studies revealed the importance of the specific 2-O HS sulfation imparted by Hs2st activity. In *C*. *elegans* Hs2st is important for cell migration while *in vitro* studies with immortalized cell lines showed that Hs2st is important for Fgf2 binding to the cell surface [15]. Consistent with the *C*. *elegans* study, HS lacking 2-O HS sulfation from mice homozygous for a gene-trap insertion in the *Hs2st* gene (*Hs2st* ^*-/-*^) showed lowered binding to Fgf2 although, puzzlingly, *Hs2st* ^*-/-*^ mouse embryonic fibroblasts *in vitro* responded normally to Fgf2 [16]. Interestingly, *Hs2st* null mutants phenocopy *Fgf2* null and Erk1/2 conditional mutants with all sharing a thinned cerebral cortex due to reduced cell proliferation providing further support to the notion that 2-O HS sulfation catalyzed by Hs2st plays a role in regulating Fgf2/Erk signaling in the developing telencephalon [12,17–19]. Nonetheless, the molecular mechanism of this interaction in this context is not fully understood. For example, 2-O HS sulfation catalyzed by Hs2st could be regulating Fgf2/Erk signaling *in vivo* by regulating the levels of the Fgf2 ligand and/or the ability of Fgf2 to bind to its receptors on the surface of target cells.

We set out to elucidate the molecular role of 2-O HS sulfation in Erk signaling *in vivo* at the telencephalic midline of E12.5 mouse by addressing each of these possibilities using *Hs2st* ^*-/-*^ mice as a model for the loss of 2-O HS sulfation. We found that 2-O HS sulfation was required for Erk1/2 activation in the telencephalic midline. Loss of signaling was not caused by reduced ligand availability as Fgf2 distribution and levels remain unchanged in the *Hs2st* ^*-/-*^ mutant compared to wild-type telencephalic midline. However we did find that 2-O HS sulfation was specifically required for the formation of HS:Fgf2:Fgfr1 ternary structure needed to activate Erk1/2. In conclusion our data suggest a model where high expression of Hs2st at the telencephalic midline produces highly 2-O sulfated HS which targets high levels of Erk signaling to the telencephalic midline by facilitating productive Fgf2:Fgfr1 signaling complex formation.

## Materials and Methods

### Animals

All mice were bred in-house in line with Home Office UK legislation. The licenses authorizing this work were approved by the University of Edinburgh Review Committee and the Home Office. Animal husbandry was in accordance with the UK Animals (Scientific Procedures) Act 1986 regulations. All mice used in the study were euthanasied using cervical dislocation.

The *Hs2st* ^*LacZ*^ (*Hs2st* ^*-*^) mutant allele was obtained by insertion of a *LacZ* gene trap into the *Hs2st* locus [20] and maintained on an inbred pigmented CBA background. *Hs2st* ^*-/-*^ embryos were obtained via crossings of heterozygous (+/*LacZ*) animals while heterozygous embryos (+/LacZ) were obtained through crossing heterozygous (+/*LacZ*) with wildtype (+/+) animals.

Genotyping of animals and embryos were performed by PCR as described previously [21].

### LacZ Histochemistry

LacZ histochemistry was done as previously described [21] but briefly, heterozygous E12.5 embryonic heads were fixed overnight in 0.2% gluteraldehyde and 100 mM MgCl_2_ at 4°C. 10 μm thin frozen sections were cut coronally using a cryostat and allowed to dry. Sections were rinsed in with LacZ wash buffer (2 mM MgCl_2_, 0.02% NP40, 0.01% sodium deoxycholate in PBS) several times and incubated with LacZ staining solution (wash buffer supplemented by 5 mM potassium ferricyanide, 5 mM potassium ferrocyanide amd 1mg/ml X-gal) overnight at 37°C.

### Disaccharide analysis

Whole telencephali were dissected from E16.5 wild-type, *Hs2st* ^*-/-*^ embryos in ice cold PBS, frozen on dry ice, and stored at -80°C prior to extraction of HS using the RIP method [22]. Individual HS samples from each embryo and HS disaccharide standards (Dextra Labs) were then subjected to BODIPY hydrazide labelling and HPLC analysis with fluorescence detection using a laser-induced fluorescence (LIF) analyser to quantify the abundance of the differently sulphated HS disaccharide species in HS from each of the samples [22–24].

Dissaccharide sulfation percentage is the measure of the proportion of sulfation in a disaccharide monomer where sulfation status could range from 0 representing an unsulfated disaccharide to trisulfated in which the disaccharide is sulfated in all sulfatable positions of 2-O, 6-O and N. It was calculated using weighted scoring of disaccharide composition according to sulfation status where sulfation in one of the position on a disaccharide molecule was weighted as 1, disulfation of a disaccharide molecule was weighted as 2 and trisulfation of a disaccharide molecule was weighted as 3. Scores was divided by 300 which is the score of maximum sulfation status. A Student’s t-test was carried out to probe for statistical significance. Only disaccharide species above detection threshold of 2% in wild-type samples were analysed.

### Immunohistochemistry

E12.5 heads were fixed in 4% PFA/PBS at 4°C overnight and 10 μm thin frozen sections were cut coronally using a cryostat and allowed to dry. For pErk1/2 immunohistochemistry, sections were boiled with 10 mM sodium citrate for antigen retrieval. After permeabilisation with 0.1% Triton-X/PBS for 5 min, sections were treated with blocking solution (20% Goat serum in 0.1% Triton-X/PBS) for 20 min followed by incubation with primary antibody rabbit anti-phospho-MAPK1/2 (Cell Signalling Technology) diluted in blocking solution at 4°C overnight. Sections were then washed with 0.1% Triton-X/PBS and incubated with goat anti-rabbit biotinylated secondary antibody (Vector Laboratories) diluted 1/200 in blocking solution. Antibody staining was visualized using a standard avidin-biotin diaminobenzidine (DAB) detection kit (Vector Laboratories). After DAB staining, sections were dehydrated and mounted in DPX. For Fgf2 immunohistochemistry, sections were permeabilised with 0.1% Triton-X/PBS for 10 min followed by treatment with blocking solution (20% goat serum in 0.1% Triton-X/PBS) for 30 min. Sections were then incubated with mouse anti-Fgf basic (Abcam) at 4°C overnight. After washing with 0.1% Triton-X/PBS, sections were incubated with goat anti mouse Alexa Fluor 488 (Invitrogen) diluted 1/200 in blocking solution. For pErk1/2 immunohistochemistry n = 5 for wild-type embryos, n = 4 for mutant embryos; for Fgf2 immunohistochemistry n = 3 for wild-type and mutant embryos.

### Western Blotting

Whole telencephalon of E12.5 brains were dissected and lysed for protein with RIPA lysis buffer (Sigma) on ice for 30 min. The lysate is then resolved on a 4–12% Bis-Tris gel (Invitrogen) and transferred to a nitrocellulose membrane (Bio-Rad). The membrane was blocked with blocking solution (Li-Cor) and incubated with primary antibody rabbit anti Fgf2 (Santa Cruz) diluted 1/500 in blocking solution at 4°C overnight. Mouse anti β-actin antibody (Abcam) diluted at 1/2,500 in blocking solution was included as protein loading control. Bound antibodies were detected by incubation for 45 min with goat anti-rabbit IgG IRDye800CW (Li-Cor) and goat anti-mouse IgG Alexa Fluor 680 (Invitrogen), both 1/12,000. All washes were with 0.1%Tween20 in PBS. Membranes were dried before scanning using an Odyssey infrared imaging system (Li-Cor). Fgf2 signal was normalized to β-actin detected in the same sample.

Control for Fgf2 antibody specificity was performed by combining rabbit anti Fgf2 antibody (Santa Cruz) with a 5-fold, by weight, excess of Fgf2 blocking peptide (Santa Cruz), made up to 500μl with PBS and incubated for 2hr. The reaction was diluted in 50% LiCor/PBS-Tween blocking buffer for incubation with Western blots. Student’s t-test was used for statistical comparison with n = 3.

### Ligand and Carbohydrate Engagement (LACE) Assay

LACE assay was performed as previously described [5,25]. Briefly, frozen sections were incubated in 0.05% NABH_4_/PBS for 15 min. After several washes in PBS, sections were incubated in 0.1M glycine at 4°C overnight. Control sections were incubated with Heparitinase I (Seikagaku) before proceeding. Sections were then treated with 1% BSA/TBS solution for 10 min before incubation with 3 μM recombinant Fgf2 (R&D Systems) and 9 μM recombinant human Fgfr1α(IIIc)-Fc (R&D Systems); 20 nM recombinant Fgf10 (R&D Systems) and 20 nM recombinant human Fgfr2β(IIIc)-Fc (R&D Systems); 30nM recombinant Fgf8b (R&D Systems) and 100 nM recombinant human Fgfr3(IIIc) (R&D Systems) at 4°C overnight. Formation of ternary structure was detected by incubation of 1/200 anti-human IgG (Fc-specific) Cy3 (Sigma) in 1% BSA/TBS. n = 5 for wild-type embryos, n = 3 for mutant embryos.

### Image Acquisition

Fluorescent images were captured with a Leica AF6000 epifluorescence microscope connected to a DFC360 camera while bright-field images were captured using a Leica DLMB microscope coupled to a DFC480 camera. Exposure settings were kept constant throughout to enable comparisons between samples. Figures were edited using Adobe Illustrator and Photoshop CS6.

## Results

### *Hs2st* ^*-/-*^ mice model specific loss of 2-O sulfation

A *LacZ* gene-trap insertion in the *Hs2st* gene results in the loss of function of the heparan sulfotransferase enzyme Hs2st [20]. As Hs2st uniquely catalyzes the sulfation of the 2-O position of the HS molecule, mice homozygous for this gene-trap insertion (*Hs2st* ^*-/-*^) were predicted to have HS lacking 2-O sulfation although the consequences for sulfation at other positions is less predictable, for example HS from *Hs2st* ^*-/-*^ mouse cultured embryonic fibroblasts had reduced 2-O sulfation and increased 6-O sulfation [16,20]. There is currently no *in vivo* data on HS extracted directly from the developing mouse telencephalon. Hence, we first analysed the HS structures of the *Hs2st* ^*-/-*^ mutant by performing a disaccharide analysis. HS was extracted from wild-type or *Hs2st* ^*-/-*^ E16.5 telencephalon to give us enough material for the sensitivity of our BODIPY HPLC detection method. The analysis revealed that while both wild-type and mutant have HS molecules, their sulfation composition differs.

**Fig 1:**
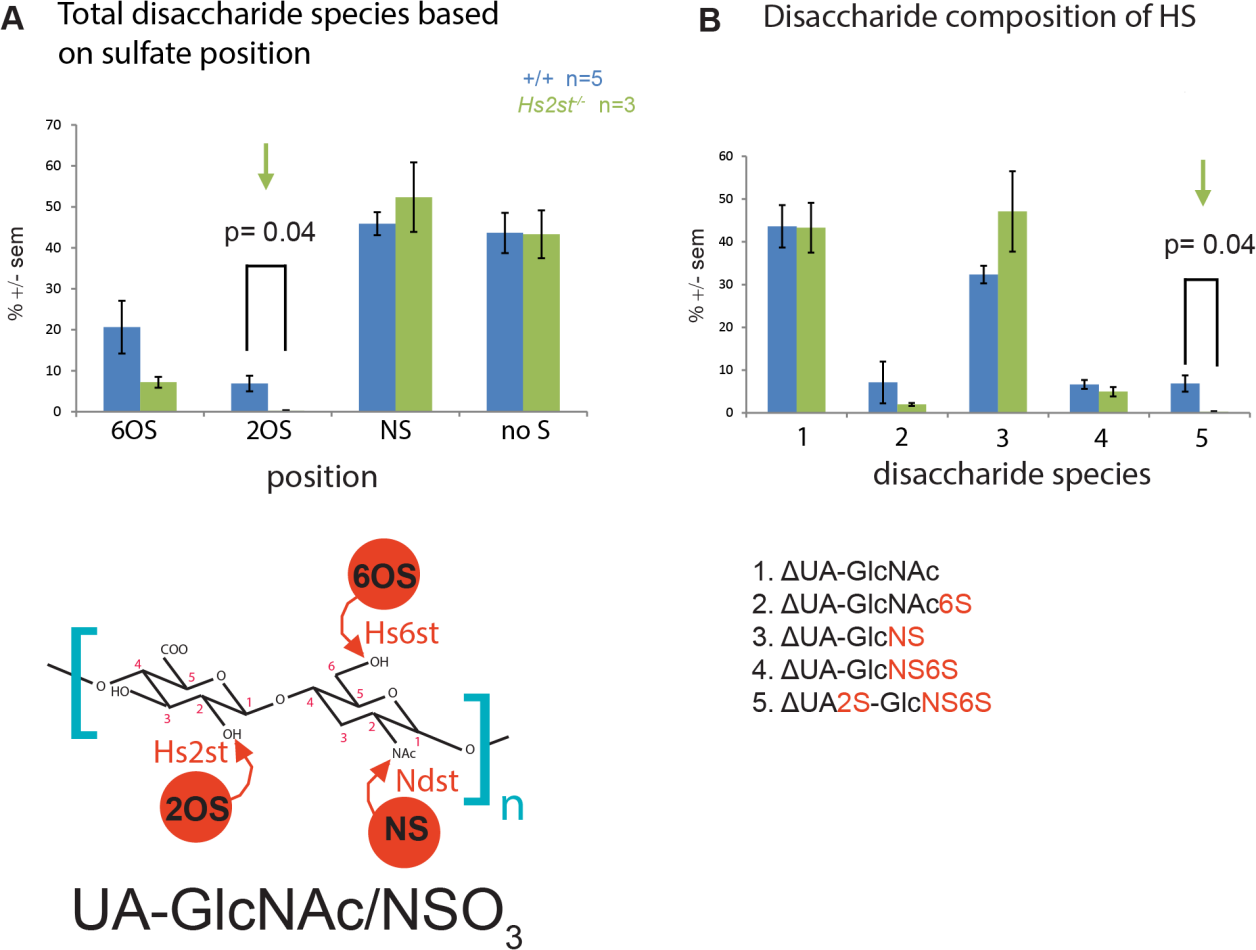
Disaccharide analysis of HS from E16.5 wild-type and *Hs2st* ^*-/-*^ telencephalon. (**A**) Proportion of sulfate occupancy in specific positions. The highest proportion of sulfate occupancy found in wild-type HS was in the N- position, followed by non-sulfated, 6-O, and 2-O positions which were 48%, 45%, 20%, and 7% respectively. HS from *Hs2st* ^***-/-***^ mutant telencephali had no significant change in the proportions of sulfate occupancy except for a significant reduction (p = 0.04) in 2-O HS sulfation in the *Hs2st* ^***-/-***^ mutant (green arrow). Diagram below graph shows position of 2-O, 6-O, or N- sulfation of the UA-GlcNAc/NSO_3_ disaccharide and corresponding HST enzyme activity. (**B**) Analysis of disaccharide composition of HS between wild-type and *Hs2st* ^***-/-***^ whole telencephalon. The only significant difference (green arrow) was the reduced trisulphated ΔUA2S-GlcNS6S disaccharide in *Hs2st* ^***-/-***^ HS (p = 0.04). There was no significant difference between wild-type and *Hs2st* ^***-/-***^ HS for other disaccharide species (ΔUA-GlcNAc, ΔUA-GlcNAc6S, ΔUA-GlcNS, ΔUA-GlcNS6S). Statistical analysis was done using ANOVA with Tukey posthoc test (wild-type, n = 5; *Hs2st* ^***-/-***^, n = 3).

In wild-type telencephali, N- sulfated disaccharides were the most abundant disaccharides at approximately 48% followed by non-sulfated disaccharides at 45%, 6-O sulfated disaccharides at 20% and 2-O sulfated disaccharides at 7% (Fig 1A–note these add up to >100% because a single disaccharide can be sulfated at more than one position). The only significant difference between wild-type and *Hs2st* ^*-/-*^ HS was a reduction (p = 0.04) in 2-O sulfation (Fig 1A, green arrow). Looking at the individual disaccharide composition of the HS in wild-type telencephali (Fig 1B), we observed that the most abundant species were unsulfated (∆UA-GlcNAc) and N-sulfated (∆UA-GlcNS) disaccharides. The most abundant O-sulfated disaccharide species were ∆UA-GlcNAc6S, ∆UA-GlcNS6S, and ∆UA2S-GlcNS6S of which only the tri-sulphated ∆UA2S-GlcNS6S contained 2-O sulfation. The remaining 2-O sulphated disaccharide species (∆UA2S-GlcNS, ∆UA2S-GlcNAc, and ∆UA2S-GlcNAc6S) were barely detectable (<2%) and we did not attempt to measure them. We saw a significant (p = 0.04) reduction in the 2-O sulfated disaccharide ∆UA2S-GlcNS6S in *Hs2st* ^*-/-*^ telencephali (Fig 1B, green arrow) while all other disaccharide species were not significantly different between wild-type and mutant (Fig 1B).

The disaccharide sulfation percentage (See Materials & Methods) provides a guide to the overall charge density of the HS molecules. We found there was no significant difference between the wild-type and *Hs2st* ^*-/-*^ disaccharide sulfation percentage values which were 24.46% ± 2.69% and 19.93% ± 2.49% (p = 0.27) respectively. Hence, we concluded that there was no major change in the overall charge density of the HS molecule in the mutant compared to the wild-type. There was also no evidence of a large compensatory increase in sulfation at other positions of the HS sugar although the average N sulfation is slightly increased, this was not statistically significant (p = 0.4, Fig 1A). Interestingly, and in contrast to previous *in vitro* findings on HS extracted from *Hs2st* ^*-/-*^ cultured fibroblasts, 6-O sulfation was not increased in *Hs2st* ^*-/-*^ telencephalic HS and in fact there was a decrease although this was not statistically significant (p = 0.19, Fig 1A). Therefore, the *Hs2st* ^*-/-*^ mice provide us a good model to study the effects of loss of 2-O HS sulfation as the *Hs2st* ^*-/-*^ mutant specifically lacks 2-O sulfation normally residing predominantly in the ∆UA2S-GlcNS6S disaccharide.

### 2-O HS sulfation correlates with Erk1/2 phosphorylation at the developing rostral telencephalic midline

**Fig 2:**
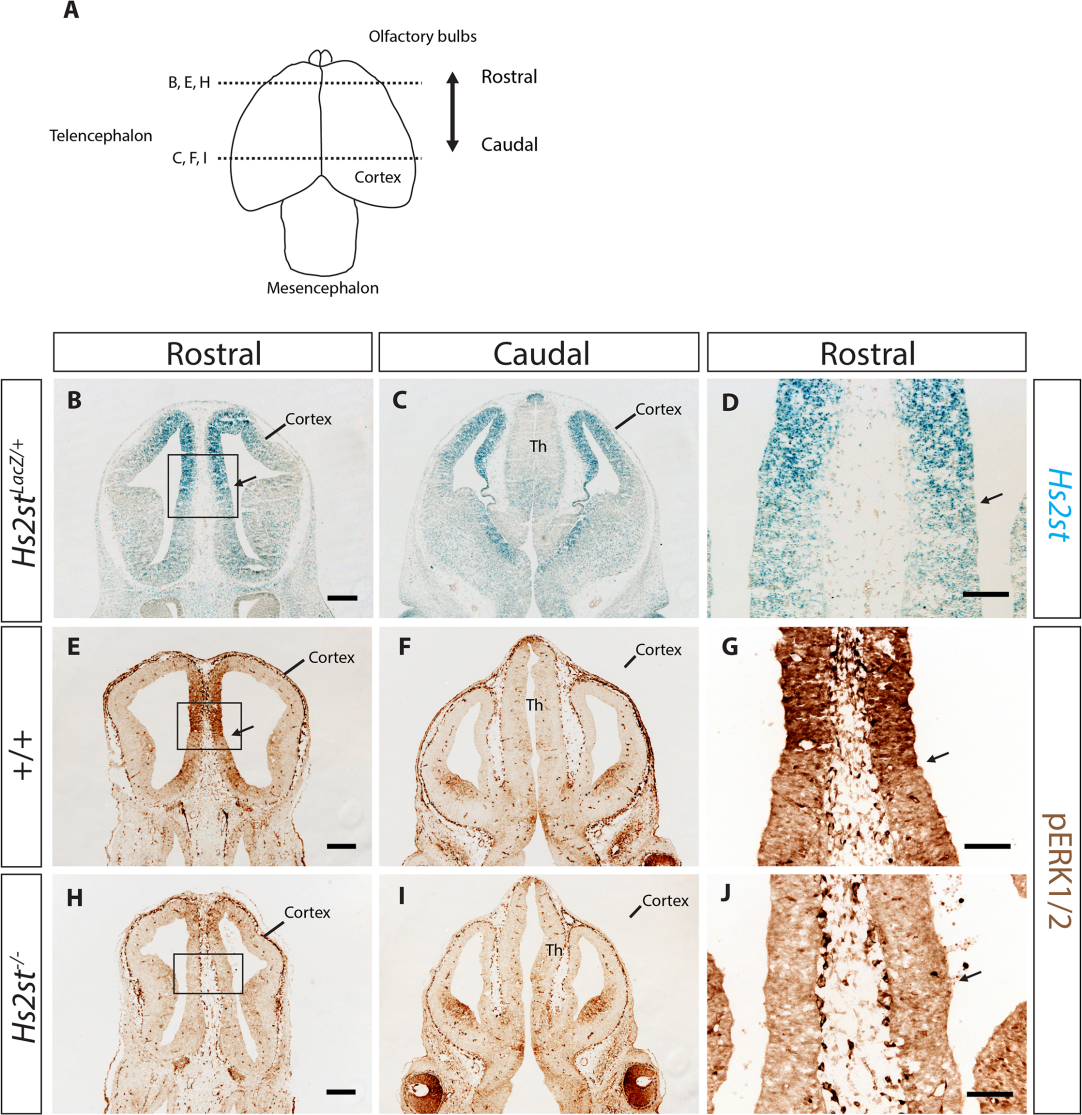
Positive correlation of Hs2st and pErk1/2 expression at the E12.5 rostral telencephalic midline. (**A**) Schematic of E12.5 brain to show the positions of coronal sections in (**B**–**J**). (**B**–**D**) *Hs2st* (blue LacZ stain) is expressed throughout the telencephalon with highest expression in the dorsal telencephalon and a clear medial^***high***^-lateral^***low***^ gradient (n = 3). (**D**) shows higher magnification of black box in (**B**). Black arrow in (**B** and **D**) show distinct border at dorsal^***high***^-ventral^***low***^ *Hs2st* expression boundary. (**E**–**G**) shows expression of pErk1/2 in wild-type telencephalon indicated by brown DAB staining (n = 5). (**E**) pErk1/2 was highly expressed in the midline. There is a distinct border between dorsal midline which has high expression of pErk1/2 and ventral midline which has low expression of pErk1/2. (**G**) is a higher magnification of black box in (**E**). Black arrows in (**E**) and (**G**) shows distinct border at dorsal^***high***^-ventral^***low***^ pErk1/2 expression boundary. (**F**) pErk1/2 was not highly expressed in the caudal telencephalon compared to the rostral telencephalon. Expression of pErk1/2 (**E**, **G**) correlates well with the expression of *Hs2st* (**B, D**) at the rostral telencephalic midline but not in the caudal telencephalon (**C, F**). (**H**–**J**) shows expression of pErk1/2 in *Hs2st* ^***-/-***^ mutants where pErk1/2 expression at the medial rostral midline was massively reduced compared to wild-type (compare **H,J** to **E,G**) but pErk1/2 expression was unchanged at the caudal telencephalon (n = 5). (**J**) is the higher magnification of black box in (**H**). Th, thalamus. Scale bars in (**B – C**, **E**–**F**, **H**—**I**) are 250 μm represented by scale bar in (**B, E, H**); scale bars for (**D, G, J**) are 100 μm.

We previously found a strong cell proliferation phenotype in *Hs2st* ^*-/-*^ telencephalon at E12.5 so we focused on this stage for our analysis of the molecular mechanism by which 2-O HS sulfation functions [17]. We used an *Hs2st LacZ* gene trap as a reporter for *Hs2st* expression to predict 2-O HS sulfation in different regions of the developing telencephalon. *Hs2st* has previously been shown to be widely expressed in the brain [12,13,17] but our new data identified a pronounced medial^high^-lateral^low^ gradient in the telencephalon at E12.5 (Fig 2B–2D) suggesting that 2-O HS sulfation is most pronounced at the telencephalic midline at this developmental stage. We also found that there was a sharp boundary separating dorsal^high^-ventral^low^ *Hs2st* expression at the rostral telencephalic midline (Fig 2B and 2D, black arrows).

Phosphorylation of Erk1/2 is one of the main molecular consequences of Fgf2 signaling and is widely used as an Fgf signaling readout [11]. Therefore, to test our hypothesis that 2-O HS sulfation plays a role in Fgf2 signaling we examined phosphorylated Erk1/2 (pErk1/2) expression in both wild-type and *Hs2st* ^*-/-*^ telencephalon. In wild-type embryos, pErk1/2 was expressed in the telencephalon where its expression was highly concentrated at the midline with a dorsal^high^-ventral^low^, and medial^high^- lateral^low^ expression pattern (Fig 2E–2G). At the rostral midline, there was a distinct border separating dorsal^high^-ventral^low^ pErk1/2 expression (Fig 2E and 2G, black arrows). However, as we move caudally along the rostrocaudal axis, we found that there was very low pErk1/2 expression at the thalamus and the caudal dorsal cortex (Fig 2F).

We noticed that the expression pattern of *Hs2st* correlates strongly with the expression of pErk1/2 in wild-type embryos at the rostral telencephalic midline (Fig 2, compare B, D with E, G) and there was a very distinct border separating dorsal^high^-ventral^low^ *Hs2st* expression correlating very well with the expression pattern of pErk1/2 (Fig 2, compare D with G, black arrows indicate border). Further caudally the expression of pErk1/2 was much weaker (Fig 2F) and we do not see a strong correlation between *Hs2st* and pErk1/2 expression (compare Fig 2C to 2F). The strong correlation between *Hs2st* and pErk1/2 expression in wild-type rostral telencephalic midline suggested the hypothesis that Hs2st expression, via 2-O HS sulfation, activates Erk signaling in the rostral telencephalic midline. We tested this by looking for reduced pErk1/2 in the rostral telencephalic midline of *Hs2st* ^*-/-*^ embryos.

There was a striking loss of Erk1/2 phosphorylation in the rostral telencephalic midline of the *Hs2st* ^*-/-*^ mutant compared to wild-type (Fig 2, compare E and H). In wild-type embryos, there was high pErk1/2 expression in the midline (Fig 2E) with a very distinct border between high pErk1/2 expression dorsally and low pErk1/2 expression ventrally (Fig 2G, black arrow). In the *Hs2st* ^*-/-*^ rostral telencephalic midline pErk1/2 expression was dramatically reduced (Fig 2H) and the distinctive dorsal^high^-ventral^low^ expression pattern was no longer apparent (Fig 2J, black arrow indicates where the border between high and low expression would be in the wild-type). However, there was no difference in pErk1/2 expression pattern between the wild-type and the *Hs2st* ^*-/-*^ mutant more caudally (Fig 2, compare F and I) suggesting that Hs2st does not play a role in Erk signaling in the caudal telencephalon. This was also supported by the fact that the expression of Hs2st and pErk1/2 do not correlate at this region in the wild-type (Fig 2, compare C and F).

Taken together, we have identified the E12.5 rostral telencephalic midline as a region where high Hs2st expression, which will likely produce high levels of 2-O HS sulfation, strongly correlates with high pErk1/2 expression. Critically the dramatic reduction of pErk1/2 expression in this region in *Hs2st* ^*-/-*^ embryos which lack 2-O HS sulfation (Fig 1) strongly suggests a causal relationship between 2-O HS sulfation and Erk signaling at the rostral telencephalic midline.

### Fgf2 protein level and distribution unaltered in *Hs2st* ^*-/-*^ mutant telencephalon

**Fig 3:**
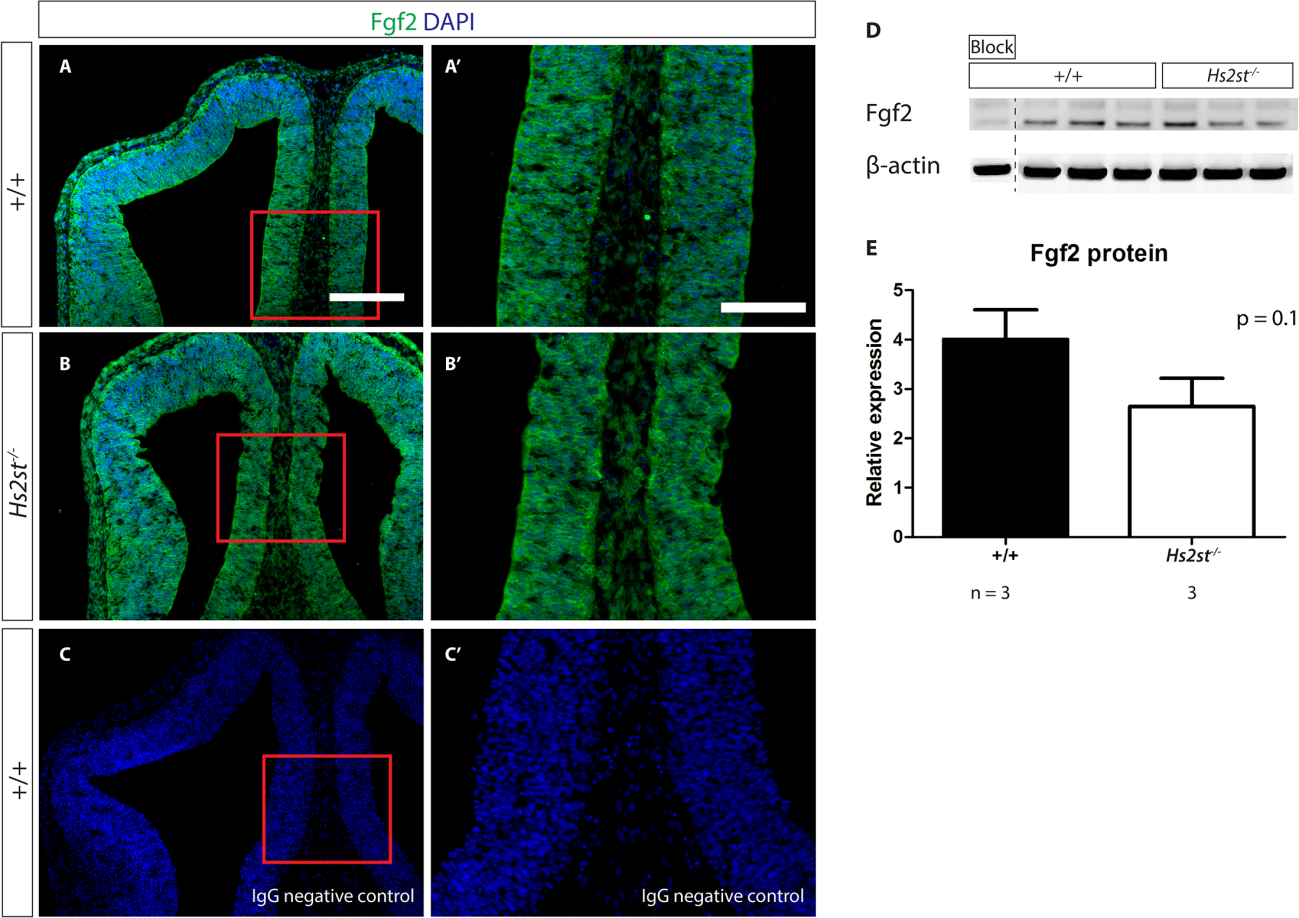
2-O HS sulfation does not affect Fgf2 protein level or distribution at the E12.5 telencephalic midline. (**A, B**) Immunohistochemistry detecting Fgf2 (green) in (**A**) wild-type or (**B**) *Hs2st* ^***-/-***^ telencephalic midline. (**C**) is the IgG negative control where primary antibody specific for Fgf2 was omitted. Nuclei labelled with DAPI (blue). (**A’**- **C’**) are higher magnification of red boxes in (**A**- **C**). (**D**) Western blot of protein extracted from E12.5 wild-type and *Hs2st* ^***-/-***^ telencephalon reacted simultaneously with Fgf2 and β-actin antibodies. Fgf2 antibody specificity demonstrated using Fgf2-specific blocking peptide (lane labelled as block). Dotted lines indicate re-arranged lanes from the same blot. (**E**) Histogram of Fgf2 protein levels in wild-type and *Hs2st* ^***-/-***^ telencephalon quantified from blot in (**D**) indicating lack of significant difference between wild-type and *Hs2st* ^***-/-***^ samples. Statistical analysis was done using Student’s T-test. Scale bars for (**A – C**) are represented in (**A)** where it is 250 μm (**A’ – C’**) are represented in (**A’)** where it is 150 μm.

The phenotypic similarity between Fgf2 and Hs2st loss of function mutant cerebral cortex suggests that Hs2st is required for normal Fgf2 function [13,17,26]. After establishing that 2-O HS sulfation plays a role in Erk1/2 activation, the downstream consequence of Fgf2 signaling, we proceeded to further dissect the molecular mechanism behind the role 2-O HS sulfation plays in this signaling pathway. The exposure of cells to the Fgf2 ligand is a critical step in activating Erk1/2 through the Fgf2 signaling pathway. HS was previously shown to stabilize several signaling ligands so we next investigated whether 2-O sulfation was affecting Erk1/2 signaling via influencing the amount of Fgf2 in the telencephalon [4,8]. This was done through the detection of Fgf2 ligand in wild-type and *Hs2st* ^*-/-*^ mutant embryos by a combination of immunohistochemistry and western blotting providing us with information about the distribution of Fgf2 and its levels respectively. Immunohistochemistry detecting Fgf2 protein showed that Fgf2 was widely and uniformly expressed throughout the telencephalon in wild-type embryos (Fig 3, A with rostral telencephalic midline shown at higher magnification in A’) as previously described [12,13]. There were no differences in Fgf2 ligand distribution between wild-type and mutant embryos (compare Fig 3A and 3A’ to 3B and 3B’). Western blot analysis of whole E12.5 telencephalon also showed there was no significant difference (p = 0.1) in Fgf2 protein levels between wild-type and *Hs2st* ^*-/-*^ mutant (Fig 3D with quantification of Western blot shown in E). Therefore, 2-O HS sulfation does not regulate the distribution or the levels of Fgf2 protein at the midline. As loss of 2-O HS sulfation in *Hs2st* ^*-/-*^ embryos does not correspond to a reduction of Fgf2 protein at the midline, 2-O sulfation is unlikely to be regulating Erk signaling via controlling Fgf2 protein concentration. Conversely, normal levels of Fgf2 in the *Hs2st* ^*-/-*^ mutant (Fig 3) are clearly not sufficient to activate Erk1/2 signaling normally in the absence of 2-O sulfation (Fig 2). One possible explanation is that 2-O sulfation is required for rostral midline telencephalic cells to respond to the Fgf2 signal.

### 2-O sulfation is specifically required for HS:Fgf2:Fgfr1 ternary structure formation at the telencephalic midline

The binding of Fgf2 ligand to Fgf receptors (Fgfr) on the cell surface initiates an intracellular signaling cascade culminating in the phosphorylation of Erk protein and abrogation of Fgfr signaling in the developing telencephalion results in reduced neuronal output[27]. Of the four Fgfrs, Fgfr1-4, Fgf2 binds most strongly to Fgfr1 and Fgfr2 *in vitro* [28]. Fgfr1 is expressed throughout the telencephalon, including high level expression at the telencephalic midline, while Fgfr2 expression is more pronounced in lateral telencephalon with very low levels at the telencephalic midline [29,30]. Taken together these data suggest the Fgf2 signal at the telencephalic midline is predominantly transduced via Fgfr1 so we next examined the sensitivity of HS:Fgf2:Fgfr1 complex formation to 2-O HS sulfation at the telencephalic midline. Previous *in vitro* studies have shown that 2-O sulfated HS is required for the binding of Fgf2 to Fgfr1 [6,7,9,10,14,16]. To date there have been no studies of this phenomenon at the midline of the developing mouse brain. We proceeded to use the ligand and carbohydrate engagement (LACE) assay, which tests the ability of exogenously added Fgf and Fgfr proteins to form a ternary HS:Fgf:Fgfr complex with endogenous HS on tissue sections, to probe whether 2-O HS sulfation is required for the formation of the HS:Fgf2:Fgfr1 ternary complex at the rostral telencephalic midline [5,25].

**Fig 4:**
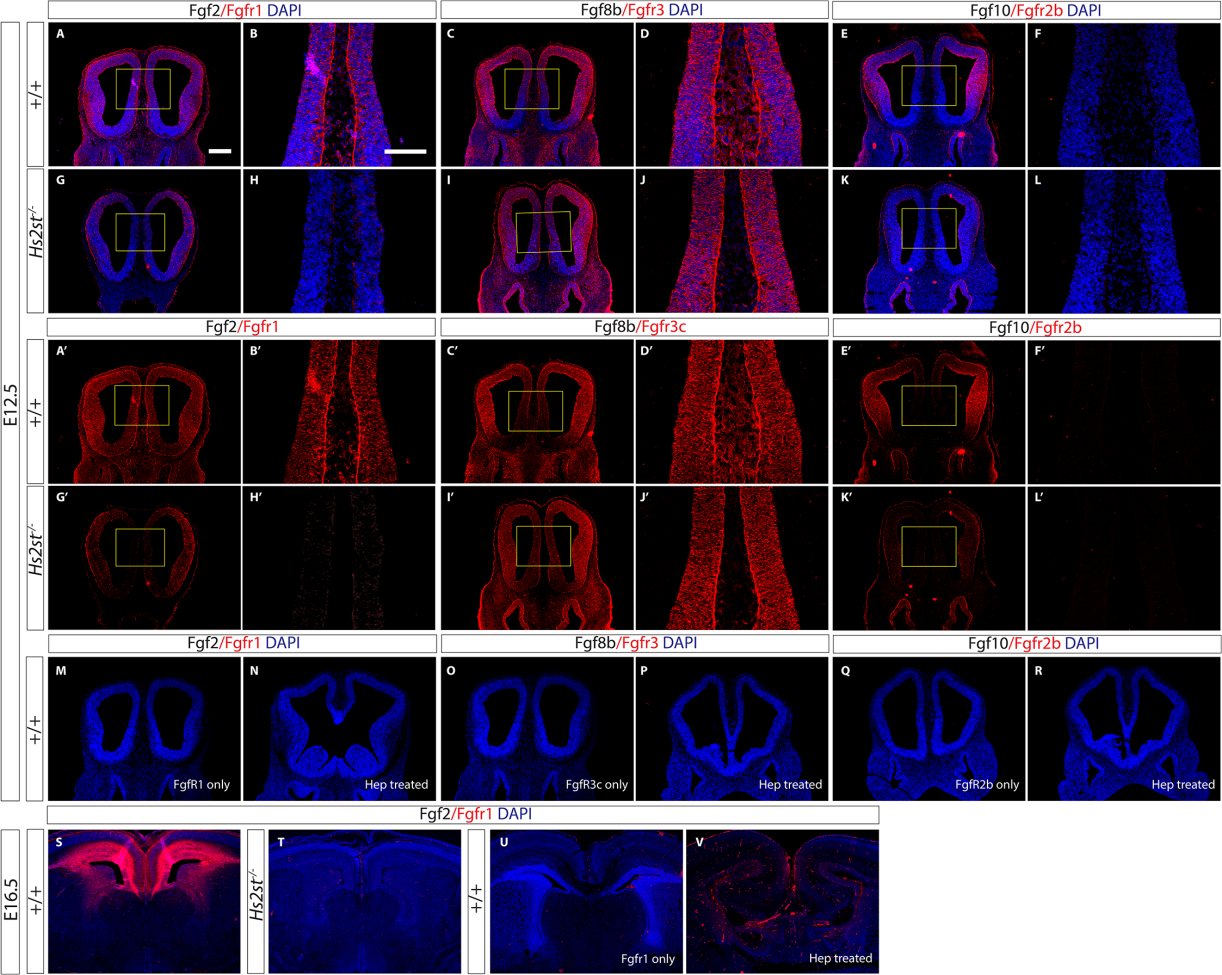
2-O HS sulfation is specifically required for the formation of HS:Fgf2:Fgfr1 ternary complexes at the E12.5 rostral telencephalic midline. (**A-V**) LACE assay probing for the ability of several exogenously added Fgf:Fgfr pairs to form complexes with endogenous telencephalic HS where LACE signal reports formation of the HS:Fgf:Fgfr ternary structure. (**A-V**) show LACE signal (red) merged with nuclear counterstain DAPI (blue) and (**A’-L’**) show LACE signal (red) alone. (**A-R**) E12.5. (**A,B,G,H,A’B’G’H’**) HS:Fgf2:Fgfr1 LACE. (**A–B, A’ – B’**) High Fgf2:Fgfr1 LACE signal throughout the wild-type telencephalon including the midline reporting formation of HS:Fgf2:Fgfr1 ternary structure. (**G – H, G’ – H’**) Very low Fgf2:Fgfr1 LACE signal at the *Hs2st* ^***-/-***^ midline compared to wild-type while LACE signal persists more laterally indicating a loss of HS:Fgf2:Fgfr1 ternary structure formation specifically in the *Hs2st* ^***-/-***^ midline. (**C – D, C’ – D’, I – J, I’ – J’)** High HS:Fgf8b:Fgfr3 LACE signal throughout the telencephalon including the midline in both wild-type and *Hs2st* ^***-/-***^ telencephalon. (**E,F,K,L,E’,F’,K’,L’**) HS:Fgf10:Fgfr2b LACE. (**E**–**F**, **E’** –**F’**) High HS:Fgf10:Fgfr2b LACE signal in the lateral wild-type telencephalon but not at the midline. (**K**–**L**, **K’** –**L’**) Low HS:Fgf10:Fgfr2b LACE signal throughout the *Hs2st* ^***-/-***^ telencephalon indicating a loss of HS:Fgf10:Fgfr2b ternary structure formation relative to wild-type in lateral *Hs2st* ^***-/-***^ telencephalon. (**B**, **D**, **F**, **H**, **J**, **L**) are higher magnification of yellow box in (**A**, **C**, **E**, **G**, **I**, **K**) respectively while (**B’**, **D’**, **F’**, **H’**, **J’**, **L’**) are higher magnification of yellow box in (**A’**, **C’**, **E’**, **G’**, **I’**, **K’**) respectively. (**S-V**) E16.5. (**S**) HS:Fgf2:Fgfr1 LACE signal in wild-type E16.5 telencephalon where high LACE signal was detected dorsally and laterally. (T) In contrast to the wild-type telencephalon, there is very little HS:Fgf2:Fgfr1 LACE signal detected in the *Hs2st* ^***-/-***^ telencephalon confirming that loss of 2-O HS sulfation disrupts the formation of the HS:Fgf2:Fgfr1 ternary structure at E16.5. Controls for the LACE assay where wild-type rostral telencephalon was reacted with Fgfr but without the respective Fgf ligand (**M, O, Q, U**) or pre-treated with Heparitinase I (**N, P, R, V**) respectively. There was no signal detected when Fgf ligand was omitted or HS was destroyed via Heparatinase I showing that the LACE assay signal reports the formation of the respective HS:Fgf:Fgfr ternary structure. Scale bars for (**C, E, G, I, K, A’, C’, E’, G’, I’, K’, M – V**) are represented in (**A**) where it is 250 μm and (**D, F, H, J, L, B’, D’, F’, H’, J’, L’**) are represented in (**B)** where it is 150 μm.

In wild-type E12.5 midline, we observed a strong signal from the LACE assay reporting the formation of the HS:Fgf2:Fgfr1 ternary complex throughout the telencephalon (Fig 4A–4A’). However, in the *Hs2st* ^*-/-*^ telencephalon the HS:Fgf2:Fgfr1 LACE signal is very weak at the midline (boxed area in Fig 4G and 4G’ shown at higher magnification in H, H’) although remains comparable to wild-type more laterally (compare lateral telencephalon in Fig 4A’ to 4G’). Therefore, the HS:Fgf2:Fgfr1 ternary structure does not form as well specifically at the midline of the rostral telencephalon when 2-O sulfation is lost. We repeated the Fgf2:Fgfr1 LACE assay at E16.5. At E16.5 Hs2st is preferentially expressed in dorsal telencephalon and ganglionic eminence and, as we saw at E12.5, the HS:Fgf2:Fgfr1 LACE signal in E16.5 wild-type embryos correlates with Hs2st expression (Fig 4S) [21]. The HS:Fgf2:Fgfr1 LACE signal is completely abolished in *Hs2st* ^*-/-*^ telencephalon (Fig 4T) demonstrating that E16.5 HS, which our disaccharide analysis formally showed has a specific loss of 2-O HS sulfation (Fig 1), is unable to support the formation of HS:Fgf2:Fgfr1 ternary complexes.

To test the hypothesis that 2-O sulfated HS is required specifically for the HS:Fgf2:Fgfr1 interaction, as opposed to a universal role in HS:Fgf:Fgfr interactions at the E12.5 telencepahlic midline, we used the LACE assay to test whether HS deficient in 2-O sulfation could still bind other Fgf-Fgfr pairs. In other contexts the formation of HS:Fgf8:Fgfr3 complex is independent of 2-O HS sulfation and HS:Fgf10:Fgfr2 complex formation is enhanced by 2-O HS sulfation so we examined these interactions in our system [5,31]. We found that HS:Fgf8:Fgfr3 complex formation was unaffected by loss of 2-O HS sulfation (compare LACE signal in Fig 4C and 4D, 4C’ and 4D’ to 4I and 4J, 4I’ and 4J’). We observed strong HS:Fgf10:Fgfr2 LACE signal in the lateral telencephalon and the interaction was much weaker medially where we were unable to detect a LACE signal (compare Fig 4E and 4F, 4E’ and 4F’ to 4K and 4L, 4K’ and 4L’). Loss of 2-O HS sulfation reduced the lateral telencephalic HS:Fgf10:Fgfr2 LACE signal showing that 2-O HS sulfation facilitates binding of Fgf10 and Fgfr2 to lateral telencephalic (but not medial telencephalic) HS suggesting that relatively low levels of Hs2st activity in the lateral telencephalon may be important for some Fgf:Fgfr signaling combinations [31].

Control LACE assays confirmed that the LACE signal reported formation of HS:Fgf:Fgfr ternary complexes as incubation with the Fgfr alone results in no LACE signal (Fig 4M, 4O, 4Q and 4U) and destroying HS with heparitinase 1 also abolishes the LACE signal (Fig 4N, 4P, 4R and 4V).

Hence, we conclude that 2-O HS sulfation is critical for the formation of HS capable of supporting the interaction between Fgf2 and Fgfr1 at the telencepahlic midline and Fgf10 and Fgfr2 in more lateral telencephalon. Conversely 2-O HS sulfation is not required for formation of the HS:Fgf8:Fgfr3 complex. Overall these data provide strong support for 2-O sulfation targeting signaling to specific ligand/receptor systems in a regionally restrained manner.

## Discussion

**Fig 5:**
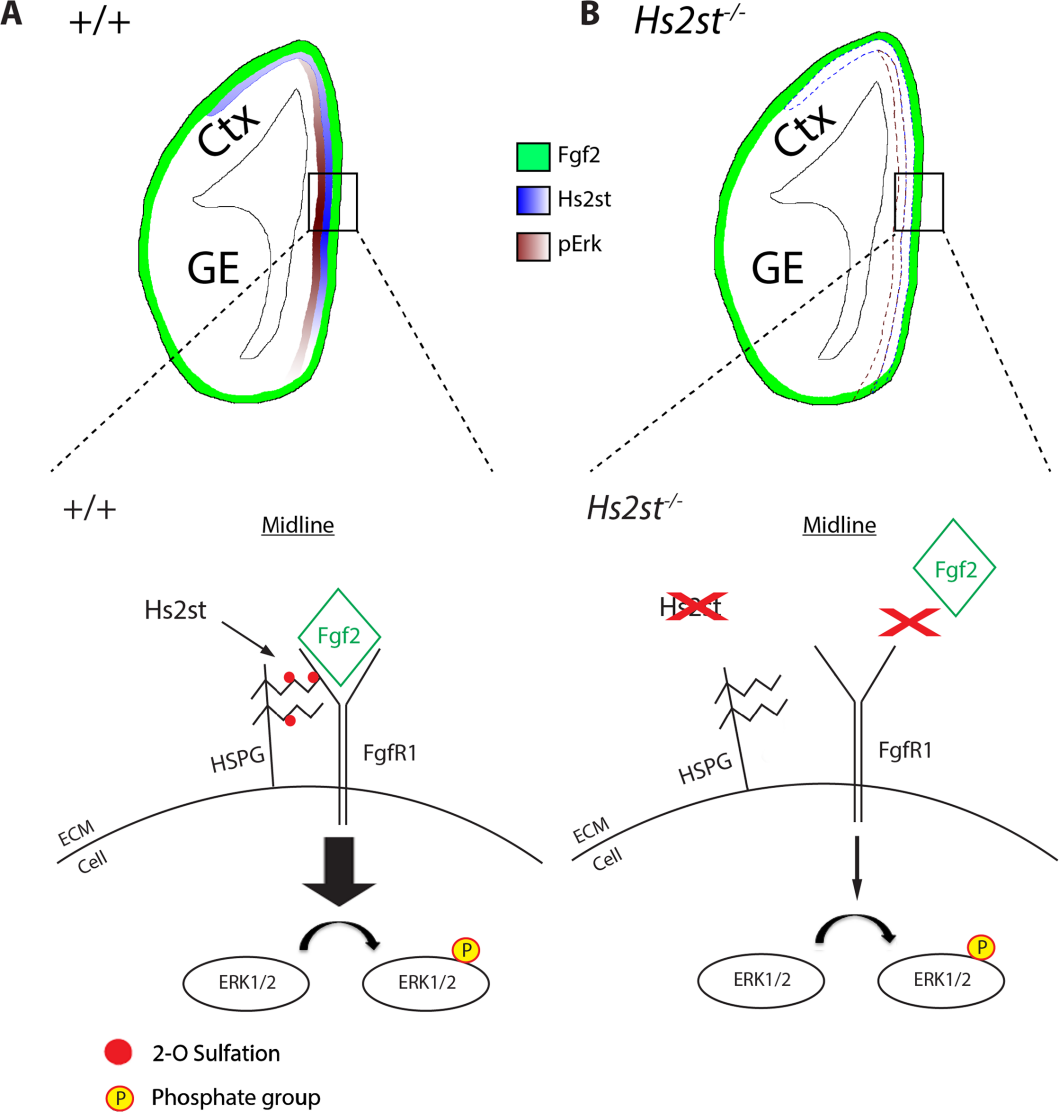
Model of how regional 2-O HS sulfation targets Fgf2/Erk signalling to the E12.5 telencephalic midline. (**A**) Top: Hs2st (blue shading) is normally regionally expressed in the telencephalon with highest levels at the midline. Fgf2 protein (green shading) is normally uniformly expressed throughout the telencephalon. pErk (brown shading) normally correlates with Hs2st with highest levels at the midline. Bottom: Hs2st catalyses 2-O HS sulfation (red disc) of the HS molecule (zig-zag line) to form a specific HS structure to allow the formation of a HS:Fgf2:Fgfr1 signaling complex which promotes the phosphorylation of Erk1/2 (**B**) Top: Without the presence of Hs2st at the midline in *Hs2st* ^***-/-***^ mice the level and distribution of Fgf2 are unaffected but pErk is dramatically reduced. Bottom: the specific 2-O sulfated HS structure that allows the formation of the HS:Fgf2:Fgfr1 signaling complex cannot form leading to a dramatic decrease of Erk1/2 phosphorylation. Thickness of arrows indicates strength of Erk1/2 phosphorylation. Ctx–cortex, GE–ganglionic eminences.

Our findings suggest a model for the regulation of the Erk signaling pathway by 2-O sulfation in the E12.5 developing mouse brain (Fig 5). The medial^high^-lateral^low^ expression of Hs2st normally results in relatively high levels of 2-O HS sulfation at the rostral telencephalic midline which in turn promotes the formation of the HS:Fgf2:Fgfr1 signaling complexes causing high Erk phosphorylation (Fig 5A). Hs2st is the only enzyme responsible for 2-O sulfation and loss of Hs2st results in the inability of cells to sulfate the HS molecule at the 2-O position as demonstrated previously [16,20,32] and for the first time in *in vivo* telencephalic tissue in the current study. HS in *Hs2st* ^*-/-*^ embryos lacks 2-O HS sulfation and is unable to form a ternary structure with Fgf2 and Fgfr1 ultimately leading to the dramatic decrease in the cells’ ability to activate Erk1/2 (Fig 5B). While some of these findings could be predicted from previous crystallography studies [9,10,33] and *in vitro* studies [6,7,14,16], to the best of our knowledge, this is the first time that 2-O HS sulfation has actually been demonstrated to be critical *in vivo* for regulating Erk signaling and the formation of HS:Fgf2:Fgfr1 complexes in neural tissue.

We find that telencephalic HS binds different Fgf:Fgfr pairs with regional specificity. HS at the midline supports the interaction of Fgf2:Fgfr1 and Fgf8:Fgfr3 but not Fgf10:Fgfr2 while lateral telencephalic HS normally supports all three Fgf:Fgfr interactions. Differential HS sulfation is important for this regional specificity as different Fgf:Fgfr pairs are differently sensitive to 2-O HS sulfation as loss of 2-O HS sulfation blocked Fgf2:Fgfr1 and Fgf10:Fgfr2 interaction while leaving Fgf8:Fgfr3 interaction unaffected. Of particular interest our study reveals that 2-O HS sulfation’s role in the regulation of Erk signaling is regionally specific in that specific HS structures rich in 2-O sulfate groups generated by Hs2st are acting as a targeting mechanism for Erk activation of at the rostral telencephalic midline. In this respect our data supports the “sugar code” hypothesis that controlled synthesis of specifically sulfated HS structures is important in regulating signaling pathways involved in brain development. There is an ongoing debate on whether HS function is conferred through the overall charge density of the HS molecule or the fine structures of the HS molecules obtained through variation of levels of sulfate positioning [1,5,34,35]. Our HS disaccharide analysis revealed that (1) the *Hs2st* ^*-/-*^ mutant telencephalon completely loses 2-O HS sulfation (2) there are no significant compensatory increase in sulfation at other positions of the HS side-chain and (3) loss of 2-O sulfation does not significantly affect overall charge density. The massive failure of HS:Fgf2:Fgfr1 complex formation in *Hs2st* ^*-/-*^ tissue therefore correlates much better with the massive loss of 2-O HS sulfation than any change in overall charge density. Taken together these data lend credibility to the idea that the position of 2-O HS sulfates rather than just their charge is the decisive factor in this context. Our analysis of HS extracted directly from embryonic telencephalon differs from analysis of HS extracted from *in vitro* cultured *Hs2st* ^*-/-*^ mouse embryonic fibroblasts in which there was evidence for compensatory increases in 6-O sulfation which were thought to restore the overall negative charge [16,36]. The difference between our current study and the previous studies could be due to differences in the source of HS analysed as the other studies assessed HS from *Hs2st* ^*-/-*^ cultured cells while we examined HS extracted directly from *in vivo* neural tissue. An interesting possibility suggested by our data is that HS sulfation *in vivo* is less subject to compensatory mechanisms than cultured non-neural cells *in vitro* which would lend support to the idea that differential expression of heparan sulfotransferase enzymes *in vivo*, perhaps particularly in developing neural tissue, can produce distinct HS structures with a greater range of biological activities.

At a later developmental stage, E16.5, we have reported that loss of 2-O sulfation has a markedly different effect on pErk1/2. At E16.5 the rostral telencephalic pErk gradient follows at medial^low^ to lateral^high^ pattern which is the opposite of the medial^high^ to lateral^low^ gradient in the E12.5 telencephalon (current study). In contrast to the dramatic decrease in pErk we observe at the E12.5 telencephalic midline in *Hs2st* ^*-/-*^ embryos, pErk levels are modestly increased at the telencephalic midline of E16.5 *Hs2st* ^*-/-*^ embryos [37]. The switch of Hs2st from positive to negative regulator of pErk between E12.5 and E16.5 could indicate that alternative signaling pathways are channeling through Erk at these two different stages, for example 2-O HS sulfation regulating Erk1/2 signaling via Fgf2 independent mechanisms [11,38,39]. Consistent with this idea Fgf2 signaling has generally been implicated in earlier developmental ages, for instance E12.5, rather than later ages, for instance E16.5, when the expression of Fgf2 has declined and Erk activation is likely to be via alternative Fgf ligands [11,12]. One plausible candidate is Fgf8 which continues to be expressed in the medial telencephalon at later stages and whose ability to form complexes with the Fgfr3 receptor is not dependent on 2-O HS sulfation (current study, [5]). Our finding that 2-O sulfation is required for formation of HS:Fgf10:Fgfr2 complexes specifically in more lateral telencephalon suggests a novel mechanism for differential HS sulfation regulating lateral cerebral cortex development via Fgf10:Fgfr2 signaling. Fgf10 is expressed transiently in cerebral cortex neural stem cells up to E12.5 and Fgfr2 is expressed in lateral telencephalon so disrupting Fgf10/Fgfr2 complex formation in *Hs2st* ^*-/-*^ cerebral cortex might impact on its development [30,40]. However, in contrast to the thinned cortex phenotype of *Hs2st* ^*-/-*^ embryos, loss of Fgf10 function expands the progenitor pool producing a thicker than normal cortex suggesting that reduced Fgf10 signaling does not make the dominant contribution to the *Hs2st* ^-/-^ phenotype ([40]).

Our data supports the notion that there is dynamism of HS structures and their biological effects on signaling pathway regulation during development. In this particular study we have shown 2-O HS sulfation regulates Erk1/2 signaling, most likely through the formation of the HS:Fgf2:Fgfr1 ternary structure required for Erk1/2 activation. Our study focused on the role of Hs2st which is just one of the enzymes producing different HS structures. Enzymes producing the other modifications, 3-O sulfation, 6-O sulfation and N-sulfation, play a part in generating HS variation but only a few are being studied [3,4]. We found 6-O sulfation by Hs6st1 normally regulates the rostral telencephalic midline glia through the suppression of Fgf8 levels at E16.5 [37], having the opposite effect on Erk1/2 to that shown in this study. Thus, manipulating HS sulfate groups in different positions has completely different effects on the Erk signaling pathway, highlighting the specificity that HS structures can provide. In addition to the binding of the ligand to its receptor as this study has shown, other aspects of signaling such as movement or level of ligand can also be regulated through HS structures [37]. All these possibilities are of significant interest and pursuing them will shed more light on the regulation of signaling pathways by HS structures.
